# Supplementary material for: Waste generation and carbon emissions of a hospital kitchen in the US: Potential for waste diversion and carbon reductions
Source: PLoS One. 2021 Mar 17;16(3):e0247616. doi: 10.1371/journal.pone.0247616 (PMC7968671; doi:10.1371/journal.pone.0247616)
Supplement: Attachment — Submitted filename: Response to Reviewers PLOS One_AAM.docx [file pone.0247616.s002.docx]

**Journal Requirements:**

**When submitting your revision, we need you to address these additional requirements.**

**1. Please ensure that your manuscript meets PLOS ONE's style requirements, including those for file naming. The PLOS ONE style templates can be found at**

[**https://journals.plos.org/plosone/s/file?id=wjVg/PLOSOne_formatting_sample_main_body.pdf**](https://journals.plos.org/plosone/s/file?id=wjVg/PLOSOne_formatting_sample_main_body.pdf) **and**

[**https://journals.plos.org/plosone/s/file?id=ba62/PLOSOne_formatting_sample_title_authors_affiliations.pdf**](https://journals.plos.org/plosone/s/file?id=ba62/PLOSOne_formatting_sample_title_authors_affiliations.pdf)

Thank you. We have modified our manuscript to meet PLOS ONE’s style requirements.

**2. We note that you have stated that the data underlying the results will be available from the corresponding author. When possible, we recommend authors deposit restricted data to a repository that allows for controlled data access. If this is not possible, directing data requests to a non-author institutional point of contact, such as a data access or ethics committee, helps guarantee long term stability and availability of data. Providing interested researchers with a durable point of contact ensures data will be accessible even if an author changes email addresses, institutions, or becomes unavailable to answer requests.  As such, we ask that you amend your Data Availability Statement to provide a durable point of contact for data requests. For more information on unacceptable data access restrictions, please see** [**http://journals.plos.org/plosone/s/data-availability#loc-unacceptable-data-access-restrictions**](http://journals.plos.org/plosone/s/data-availability#loc-unacceptable-data-access-restrictions)

**In your revised cover letter, please address the following prompts:**

**a) If there are ethical or legal restrictions on sharing a de-identified data set, please explain them in detail (e.g., data contain potentially sensitive information, data are owned by a third-party organization, etc.) and who has imposed them (e.g., an ethics committee). Please also provide contact information for a data access committee, ethics committee, or other institutional body to which data requests may be sent.**

**b) If there are no restrictions, please upload the minimal anonymized data set necessary to replicate your study findings as either Supporting Information files or to a stable, public repository and provide us with the relevant URLs, DOIs, or accession numbers. For a list of acceptable repositories, please see** [**http://journals.plos.org/plosone/s/data-availability#loc-recommended-repositories**](http://journals.plos.org/plosone/s/data-availability#loc-recommended-repositories)**.**

**We will update your Data Availability statement on your behalf to reflect the information you provide.**

We have uploaded the raw data points in spreadsheet form to be used as supplemental information.

**Reviewer Comments:**

**Reviewer #1**

**The manuscript technically sound and authors made a good attempt to quantize the food waste generated in a hospital kitchen of US and will be very helpful for the society to take actions to reduce the amount of food waste and utilize the generated food waste for energy production and composting purposes which will also help to reduce GHG emission. As author already mentioned that the study is based on one day audit, if it will be elaborated for long time with more results, i.e., characterization of food waste for its utilization for energy generation, the research will be more fruitful for the society.**

Thank you for this feedback. We would love to be able to conduct longer-term waste audits to provide a more thorough understanding of food waste in New York City in the future. Our quantification of annual hospital waste and estimation of GHG emissions in this study is a first step to building a foundation for that research.

**I want to suggest some minor corrections which will help the author to make the manuscript more fit to publish in PLOS ONE:

1. 75% + should be written as 'more than 75%' (line 87) and 90%+ should be written as 'more than 90%' (line 88).**

We have modified the text as you described.

**2. Are terms 'all day audit' (line 143) and 'detailed audits' (line 145) given by author? or if reported before than reference should be added.**

We created these terms for ease of use throughout the paper. We included them in quotation marks in lines 143 and 145 to show that we would be using these terms in the paper, and provided the definition in these lines to help the reader understand what we would be referring to.

**Reviewer #2:**

**1. Introduction could be more concise to make it easy for understanding. A lack of flow between the sentences are observed. Too much literature cited but lack of deep discussion observed.**

We have added more explanatory information to the introduction and eliminated citations of literature that distracted from main points. We feel the introduction is now more concise and better informs the reader—thank you for your feedback.

**2. Research highlights could be added that include main findings instead of general statements.**

We currently state the main findings in the abstract and in the first paragraph of the discussion. We have now elaborated on our main findings in the abstract and discussion and added main findings to the conclusion as well (new text highlighted in yellow below):

Abstract, page 2:

Extrapolating to all meals served in 2019, the hospital kitchen generates over 442,067 kg (487 US tons) of waste and emits approximately 294,466 kg of CO_2_e annually from waste disposal. Most of this waste (85%, 376,247 kg or 415 US tons annually) is currently sent to landfill. With feasible changes, including increased recycling and moderate composting, this hospital could reduce landfilled waste by 205,245 kg (226 US tons, or 55% reduction) and reduce GHG emissions by 189,025 kg CO_2_e (64% reduction).

Discussion, pages 12-13:

In this study, we found that a single hospital in NYC annually generates 442,067 kg of waste (487 US tons) and emits approximately 294,466 kg of CO_2_e from waste disposal. The majority of this waste (85%, 376,247 kg or 415 US tons annually) is currently sent to landfill. If the hospital were to increase recycling rates and implement composting in a feasible manner, it could reduce landfilled waste by 205,245 kg (226 US tons, or 55% reduction) and GHG emissions by 189,025 kg CO_2_e (64% reduction). Although an ideal waste diversion scenario would divert 76% of waste from landfill and reduce GHG emissions by 90%, it would be particularly challenging to achieve due to available commercial composting facilities, vendor restrictions, staffing resources, space constraints, logistics, and expenses.

Conclusion, page 16:

Hospital kitchens and other institutional food services generate a substantial amount of solid waste and greenhouse gas emissions. However, there are ample opportunities to reduce and divert this waste from methane-producing landfills. Using waste audit methods at a case location, we found that the hospital kitchen in this study annually sends 85% of its waste—415 US tons—to landfill, but that it could feasibly reduce its waste to landfill by 55% and subsequently reduce its GHG emissions by 64%. This study provides a valuable framework from which hospitals and policymakers in NYC and elsewhere can begin to measure and subsequently reduce institutional food waste. It is also particularly timely, as NYC’s OneNYC strategic plan has targeted sustainability goals including Zero Waste by 2030 and carbon neutrality by 2050.

**3. Please update your work by comparing the latest articles published in journals.**

There are very few published articles to compare our results with, as this topic is not highly studied. However, we added a new citation to the discussion (page 13, new text highlighted in yellow below):

Despite a dearth of data on waste diversion in other hospitals, one case study of a 250-bed hospital in San Francisco estimated that an institutional waste diversion program could result in an annual reduction of 51 US tons of landfilled waste. That estimate is lower than the one found in this study, which is likely due to differences in the size of the hospitals’ patient populations and foodservice operations, and variations in waste diversion strategy design.

Freedman MR, Franklin IB. Implementing a solid waste management diversion program in a conventional cook–serve hospital system: A feasibility study. J Hunger Environ Nutr. 2010 Aug 31;5(3): 370-9.

**4. In scientific publication reference of website is not acceptable e.g US EPA. Greenhouse Gas Equivalencies Calculator 2018.**

According to PLOS ONE’s submission guidelines at https://journals.plos.org/plosone/s/submission-guidelines#loc-references, published media (print or online newspapers and magazine articles) and new media (blogs, websites, or other written works) are acceptable to cite and include in the reference list. The only sources PLOS ONE instructs not to cite in the reference list are unavailable and unpublished work and personal communications. We have thus retained references to websites, but if the editor and reviewers feel it is necessary to remove website references, we will do so.

**5. Some of the references seems incomplete like Mirosa M, Munro H, Mangan-Walker E, Pearson D. Reducing waste of food left on plates. British Food Journal. 2016. There are more**

Thank you for highlighting this. We have reviewed and modified our references to ensure they are properly formatted for PLOS ONE. For example, we changed the reference you highlighted to: Mirosa M, Munro H, Mangan-Walker E, Pearson D. Reducing waste of food left on plates. Br Food J. 2016;118(9): 2326-2343.

**6. Results lacks statistical analysis**

As we discuss in the methods section (page 7), our paper uses descriptive statistics to characterize the results of our waste audits. We then use those results to quantify annual waste generation and estimate greenhouse gas emissions from three waste diversion scenarios. We did not include other statistical methods because the goal of this study was to provide descriptive data. We believe this study provides a valuable framework from which hospitals and policymakers can begin to measure and subsequently reduce institutional food waste.

**7. Limitations itself says the story of data, as Variability obviously exists by day of the week, season, and year, and by the way in which a hospital’s food service operate. Such study requires long term study. Study is very concise.**

We understand that this study only shows waste from one hospital kitchen on a single day, and that longer term studies are needed. However, we believe this study creates an important foundation that can be used to design and implement longer-term studies. We have added text to make that clearer in the limitations section (page 16, new text highlighted in yellow below):

This study analyzed only one hospital kitchen on a single day. Variability obviously exists by day of the week, season, and year, and by the way in which a hospital’s food service operates. Additionally, this study did not capture staff and visitor waste from retail meals, as the cafeteria’s front-facing waste stream does not come through the kitchen. As a result, this study underestimates the hospital’s total food service wastes and potential diversion opportunities. In addition, this study used a sampling frame to estimate the composition of daily kitchen wastes. This study also utilized GHG factors from the US EPA’s WARM model, which contains its own assumptions about landfilling, recycling, and composting. Other GHG models may estimate emissions differently. Future studies should audit waste in detail over a longer time period (e.g., multiple days throughout the year), and further investigate opportunities to reduce staff and visitor cafeteria waste. Future studies should also investigate other opportunities for organic waste diversion, including anaerobic digestion, and calculate and compare costs of various waste diversion strategies, including composting, anaerobic digestion, and recycling.

**Reviewer #3: The manuscript titled " Waste generation and carbon emissions of a hospital kitchen in the US: potential for waste diversion and carbon reductions" by Thiel et al., did a survey of waste generation from NYC hospital from New York, categorized the biodegradable and recyclable fractions, quantified and calculated the GHG emission contributions by its current disposal practices. The study also proposed few alternate options to recycle the waste as compost and reduce the burden of landfill operation in future.**

**The study is very basic and concluded based on one singe data set, which is not realistic. The composition and quantity of the waste might not be the same throughout the year, while the study doesn't include any scientific elements.**

We understand that this study only shows waste from one hospital kitchen on a single day, and that longer term studies are needed. However, we believe this study creates an important foundation that can be used to design and implement longer-term studies. We have added text to make that clearer in the limitations section (page 16, new text highlighted in yellow below):

This study analyzed only one hospital kitchen on a single day. Variability obviously exists by day of the week, season, and year, and by the way in which a hospital’s food service operates. Additionally, this study did not capture staff and visitor waste from retail meals, as the cafeteria’s front-facing waste stream does not come through the kitchen. As a result, this study underestimates the hospital’s total food service wastes and potential diversion opportunities. In addition, this study used a sampling frame to estimate the composition of daily kitchen wastes. This study also utilized GHG factors from the US EPA’s WARM model, which contains its own assumptions about landfilling, recycling, and composting. Other GHG models may estimate emissions differently. Future studies should audit waste in detail over a longer time period (e.g., multiple days throughout the year), and further investigate opportunities to reduce staff and visitor cafeteria waste. Future studies should also investigate other opportunities for organic waste diversion, including anaerobic digestion, and calculate and compare costs of various waste diversion strategies, including composting, anaerobic digestion, and recycling.

Regarding scientific elements, the goal of this study was to provide descriptive data, so we utilized descriptive statistics to characterize the results of our waste audits (see methods, page 7). We then used those results to quantify annual waste generation and estimate greenhouse gas emissions from three waste diversion scenarios. We believe this study provides a valuable framework from which hospitals and policymakers can begin to measure and subsequently reduce institutional food waste.

**Composting process also emit lot of GHGs (N2O and CO2) and this would be more than landfill based GHG emissions that is calculated in this study.**

As we describe in our methods on page 8, we used the US EPA’s Waste Reduction Model (WARM) to estimate GHG emissions from waste (including landfill and composting). The US government developed this model to help solid waste planners and organizations track and voluntarily report GHG emissions reductions, energy savings and economic impacts from several different waste management practices. The net GHG emissions from composted material in this model are negative, as explained by the EPA in their WARM manual (https://www.epa.gov/sites/production/files/2019-10/documents/warm_v15_management_practices_updated_10-08-2019.pdf):

“As modeled in WARM, composting results in some carbon storage (associated with application of compost to agricultural soils), carbon dioxide (CO2) emissions from transportation and mechanical turning of the compost piles, in addition to fugitive emissions of methane (CH4) and nitrous oxide (N2O) produced during decomposition. To estimate the carbon storage from compost application, EPA selected point estimates from the range of emission factors covering various compost application rates and time periods. EPA chose the point estimates based on a typical compost application rate of 20 short tons of compost per acre, averaged over four soil-crop scenarios. EPA selected the carbon storage values for the year 2010 to maintain consistency with the forest carbon storage estimates discussed in the Forest Carbon Storage chapter. Overall, EPA estimates that centralized composting of mixed organics results in net carbon storage of 0.18 MTCO2E per wet short ton of organic inputs composted and applied to agricultural soil.”

We understand that other models may estimate emissions differently, and have thus included the following limitation in our discussion section (page 16):

This study utilized GHG factors from the US EPA’s WARM model, which contains its own assumptions about landfilling or composting. Other GHG models may estimate emissions differently.

**Overall the paper required more detailed study and data collection, interpretations to be accepted for publication.**

We provide detailed information about our study design and data collection in the methods section (pages 4-8). We have revised the order of the calculation of waste diversion scenarios and greenhouse gas emissions to provide a clearer understanding for the reader in both the methods and results sections. As for including more detailed interpretations, we have now added a comparison of our results in the discussion to results found in previously published waste audits, so that our manuscript references more of the existing literature (page 13, new text highlighted in yellow below):

Despite a dearth of data on waste diversion in other hospitals, one case study of a 250-bed hospital in San Francisco estimated that an institutional waste diversion program could result in an annual reduction of 51 US tons of landfilled waste. That estimate is lower than the one found in this study, which is likely due to differences in the size of the hospitals’ patient populations and foodservice operations, and variations in waste diversion strategy design.

Despite structural differences between hospitals, diverting organic and recyclable hospital waste from landfills could be highly impactful if implemented across NYC, as the city is home to 41 hospitals and 15,841 beds.

Freedman MR, Franklin IB. Implementing a solid waste management diversion program in a conventional cook–serve hospital system: A feasibility study. J Hunger Environ Nutr. 2010 Aug 31;5(3): 370-9.

**Composting process is not ideal food waste, instead anaerobic digestion of food waste and energy recovery could be considered as a option. Cost and GHG emissions from the food waste processing by landfilling, composting and anaerobic digestion need to be compared and discussed.**

Composting is a common method of treating food waste, especially in New York City, where this study took place. Anaerobic digestion/energy recovery is another option for treating organic material though—while we did not model that option, it is clearly an area for future research. We also did not incorporate cost into our study, as that was outside the scope of our research, but it is an area for future research as well. We have modified various sections of the discussion to address these points (new text highlighted in yellow below):

Discussion, page 15:

Finally, food and recyclable wastes should be diverted from landfills, either through composting, recycling, or anaerobic digestion, which uses microorganisms to break down organic material into biogas, which can be used as a renewable energy source. Substantial barriers exist to implementing these strategies, however. Many hospitals need to establish contracts with private or commercial composters/anaerobic digestion operators, and for many, recycling is a much more commonly implemented waste diversion initiative [35]. Cities might consider supporting local composting infrastructure, and enacting legislation that encourages large institutions to utilize these waste streams. For example, prior to COVID-19, the NYC Department of Sanitation (DSNY) offered composting in residential areas and for non-profit organizations. DSNY also partners with a non-profit called GrowNYC that facilitates food scrap drop-off sites in local farmers markets.

Limitations (Discussion), page 16:

Future studies should also investigate other opportunities for organic waste diversion, including anaerobic digestion, and calculate and compare costs of various waste diversion strategies, including composting, anaerobic digestion, and recycling.
